# Deformation characteristics of overlying strata in room and pillar mined-out areas under coal pillar instability

**DOI:** 10.1038/s41598-023-50996-3

**Published:** 2024-01-10

**Authors:** Yaming Liu, Tianfeng Gu, Yanchao Wang, Wei Xiong, Xuanyu Yang

**Affiliations:** 1 Shanxi Province Transportation Technology Research and Development Co., Ltd., Taiyuan, 030032 Shanxi People’s Republic of China; 2 Shanxi Highway Safety Transportation Technology Co., Ltd., Taiyuan, 030800 Shanxi People’s Republic of China; 3 State Key Laboratory of Continental Dynamics, Department of Geology, Northwest University, Xi’an, 710069 Shaanxi People’s Republic of China

**Keywords:** Environmental impact, Civil engineering

## Abstract

Under the condition of small roof deformation before the occurrence of fractures and collapse in room and pillar mined-out areas caused by coal pillar instability, the surface deformation may be large, which threatens the safety of ground structures. Interferometric synthetic aperture radar, geophysical exploration, geotechnical exploration and physical simulation tests were conducted to analyse the deformation and development mechanism of the overlying strata in the mined-out area in this case. The results show that in terms of surface deformation, the surface deformation caused by coal pillar instability in the room and pillar mined-out area exhibits the slow deformation stage, uniform deformation stage and accelerated deformation stage. In terms of deformation of overlying strata, after the completion of room and pillar mining, a strip-shaped deformation area and trapezoidal deformation area are developed in the overlying rock. With the occurrence of coal pillar instability, a trapezoidal deformation area and inverted funnel-shaped deformation area are developed in the overlying rock. The deformation characteristics of unconsolidated formations transition from trapezoidal deformation after room and pillar mining to funnel-shaped deformation due to coal pillar instability. Moreover, the maximum surface deformation point is located at the centre of the funnel. In terms of spatial morphology of mined-out area deformation, the maximum surface deformation point corresponds to the position of the initial coal pillar instability and the crack in the mined-out area roof along the vertical direction. The mined-out area treatment method can be optimized based on the deformation characteristics of the overlying strata in the room and pillar mined-out area under the condition of coal pillar instability.

## Introduction

Mined-out areas, which are cavities formed after the mining of mineral resources, are widely distributed in coal resource-rich areas. Ground subsidence caused by underground mining has a great influence on the safe operation of infrastructures, such as buildings, roads and railways, and can even cause the failure of structures. From the 1980s to the early twenty-first century, the village-run collective coal mines and private coal mines in China's main coal-producing areas mostly mined coal resources through the room and pillar coal mining method, resulting in many room and pillar mined-out areas remaining in China's old mining areas. The main deformation of the mined-out area with a high mining rate formed by longwall mining generally lasts 1–3 years. Because of the coal pillar left in the room and pillar mined-out area, the stability of the roof can be better ensured, and the roof can be prevented from cutting off. However, under the long-term effects of groundwater and coal pillar weathering, creep failure and the instability of coal pillars occur, and room and pillar mined-out areas often collapse decades after the end of coal mining, even leading to the occurrence of mine earthquakes^1–7^.

The existing mining subsidence theory is based on the high mining rate of longwall mining. In the process of coal mining, the support of the working face close to the mined-out area is withdrawn so that the roof collapses, leading to the movement, deformation and failure of the overlying strata. With the increase in the advancing distance of the working face, overburden failure develops upwards and causes surface movement, forming a surface movement basin^8–15^. In the mined-out area with a low mining rate formed by room and pillar mining, the deformation law of the overburden is different. Under the support of a coal pillar, the room and pillar mined-out area roof can remain stable for a long time. In the current study, the buckling deformation of the room and pillar mined-out area is established under the condition of coal pillar instability. Currently, the low mining rate and insufficient mining movement result in a high mining rate, and full mining movement and roof breakage occur, resulting in overlying strata deformation. The deformation law of overburden rock is consistent with the high mining rate of the longwall mining rock deformation law^2^. When the partial instability of the coal pillar causes the small deformation of the roof before fractures and collapse, it is in the intermediate state of a low mining rate and insufficient mining movement to a high mining rate and full mining movement. It is generally believed that the overlying strata are in a stable state, and the overlying strata are in the stage of elastic or plastic deformation, which does not cause the deformation of the unconsolidated formation^2,16^. However, in some special geological structures, such as the large thickness of the unconsolidated formation and weak overlying rock above the roof, small deformations of the roof before the fractures and collapse may lead to large deformation of the surface and affect human activities on the ground, which has not been given enough attention in previous studies.

In recent years, research on mining subsidence theory has mainly focused on surface deformation in mined-out areas. Mancini et al.^17^ analysed surface deformation in a mined-out area in Bosnia and Herzegovina using historical terrain data and evaluated the risk of surface deformation. Kelly et al.^18^ studied geological and mechanical problems in longwall mined-out areas and developed a method for evaluating stability in longwall mined-out areas. Based on the coal mining method, depth, mining sequence, and other geological and mechanical characteristics, Unlu et al.^19^ proposed a new settlement prediction method (ISP-Tech) to study ground subsidence caused by underground mining activities. Salmi et al.^20^ studied the impact of the gradual deterioration in rock masses on the settlement mechanism of shallow abandoned coal mines. Ghabraie et al.^21^ proposed a prediction method for the induced surface subsidence in a multiseam mined-out area.

In the study of the mechanism of geological disasters in mined-out areas, indoor experiments, numerical simulations, on-site monitoring and other methods have been used^22–31^. During indoor testing for investigating mined-out areas, similar materials have been selected for layered masonry simulation of different strata, mainly studying the deformation characteristics of the overlying rock in mined-out areas, with less consideration given to the deformation of loose layers^22,24,25,27,30^. In numerical simulation of mined-out areas, the discrete element method has often been employed, but the simulation results are greatly influenced by the calculation model and rock mechanics parameters^23–25^. During on-site monitoring of deep deformation in mined-out areas, sensors are often installed in deep boreholes to observe the deformation of rock layers directly or indirectly. With the use of the point monitoring method, it is difficult to capture the overall deformation of the overlying strata in the mined-out area^23^. In the study of the deformation mechanism of the overlying strata in shallow buried room and pillar mined-out areas, Yang et al.^29^ proposed a classification method for mining loose layer structures based on the geological conditions in the Shendong mining area and constructed a mechanical model of the arch beam.

In this article, we conduct physical simulation experiments based on particle image velocimetry (PIV) image processing technology and modular assembly ideas. The evolution of the entire displacement field of the overlying strata in the mined-out area reflects the development mechanism of goaf disasters under the condition of coal pillar instability, whereby the roof in the room and pillar mined-out area is deformed but does not collapse. The research results provide a certain reference significance for disaster prevention and reduction and environmental protection of projects in room and pillar mined-out areas.

## Study area

**Figure 1:**
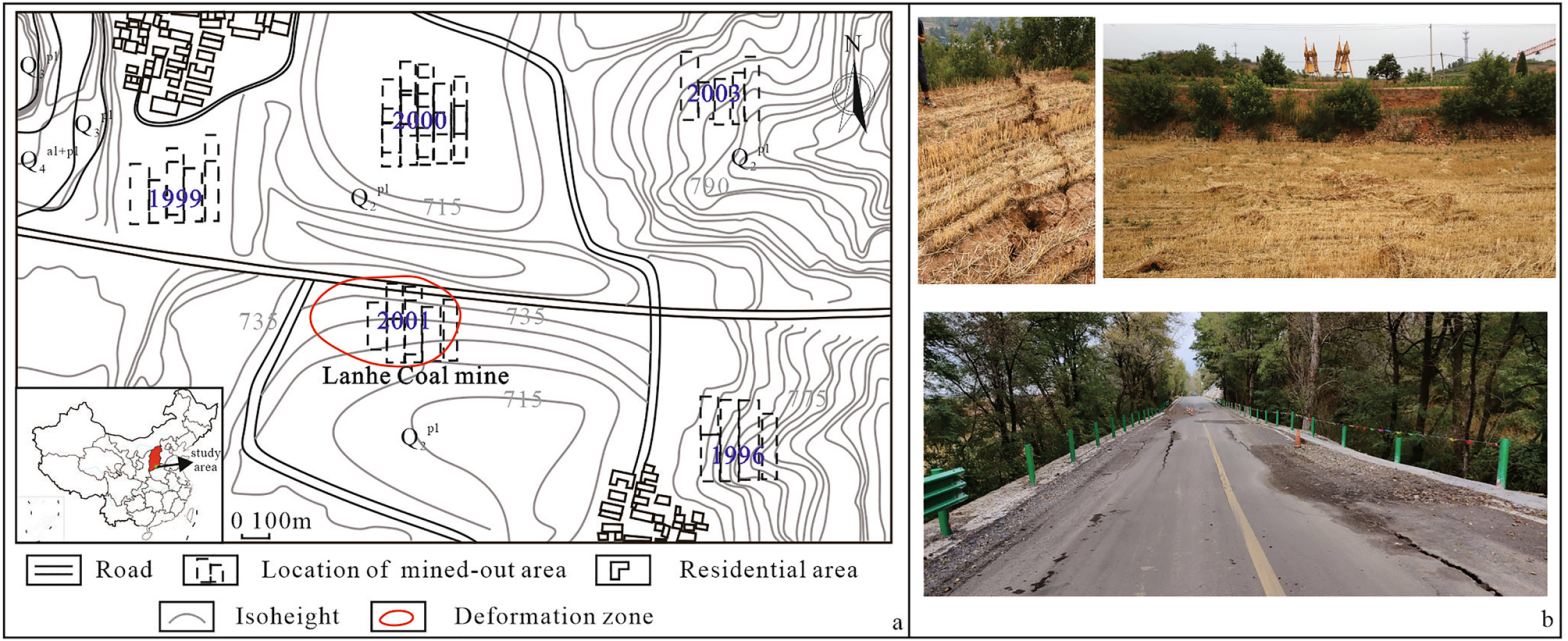
Location of the study area.

The study area is located in Jincheng city, Shanxi Province, China (Fig. 1a). It is located in the middle mountainous area covered by Quaternary unconsolidated formations on the west side of the southern section of Taihang Mountain and the southeast side of the Qinshui coalfield. The coal seams that can be mined in this area are mainly the #9 and #15 coal seams of the Upper Carboniferous Taiyuan Formation and the #3 coal seam of the lower Permian Shanxi Formation. In the 1990s and early 2000s, many room and pillar coal mined-out areas were generated in this area (Fig. 1a).

The Lanhe Coal Mine is a village-run coal mine, mainly mining the #3 coal seam of the lower Permian Shanxi Formation, with a field area of 0.185 square kilometres and a geological reserve of 1.66 million tons. Its design production capacity, which adopts the inclined shaft development method, is 30,000 tons/year. The coal mine adopts room and pillar mining, the mining width and coal pillar width are approximately 7 m, the mining rate is 50%, the burial depth is approximately 50 m, and the average mining thickness is 5 m. Moreover, this mine was closed in 2001.

Nearly 20 years after the end of the room and pillar mining, the surface is basically in a stable state, and no cracks have developed. In August 2020, local residents found that large surface deformation occurred above the goaf of the Lanhe Coal Mine, leading to road cracking, road surface subsidence and damage, large-scale farmland subsidence and the development of many cracks (Fig. 1b), which had a great impact on road safety operation and agricultural ecology.

## Methodology

In this paper, interferometric synthetic aperture radar (InSAR), geophysical exploration and geotechnical exploration, and physical simulation tests are used to study the cause and development mechanism of surface deformation above the room and pillar mined-out area in the Lanhe Coal Mine.

**Figure 2:**
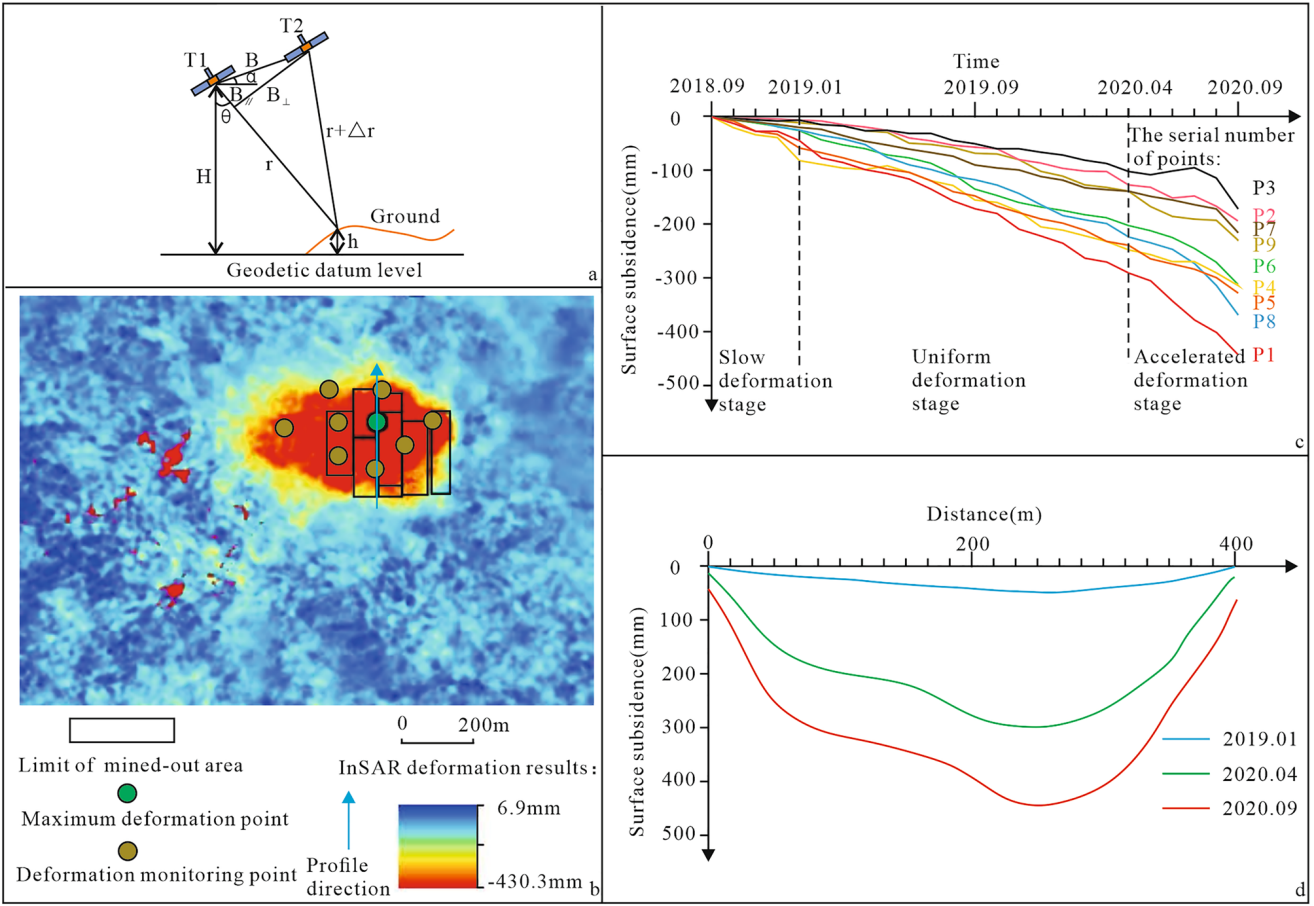
InSAR monitoring chart.

To obtain the law of surface deformation, small baseline subset (SBAS)-InSAR (Fig. 2a)^32^ was used to identify and monitor the surface deformation area in the study area with the wide-amplitude interference data of ESA Sentinel 1A. The spatial resolution of the data was 5 m × 20 m (range direction × azimuth direction). The data span is from September 2018 to September 2020, and the number of images is 25, with a time interval of 24 or 36 days to ensure one scene per month. The auxiliary digital elevation model (DEM) in the calculation is the SRTM3 data provided by NASA, with a ground resolution of 3 arcseconds and an elevation accuracy of approximately ± 16 m, which is used to remove the terrain phase in the interferogram. In addition, to improve the computational efficiency, the whole image is clipped and then calculated, setting spatial and temporal baseline thresholds of 200 m and 500 days, respectively.

**Figure 3:**
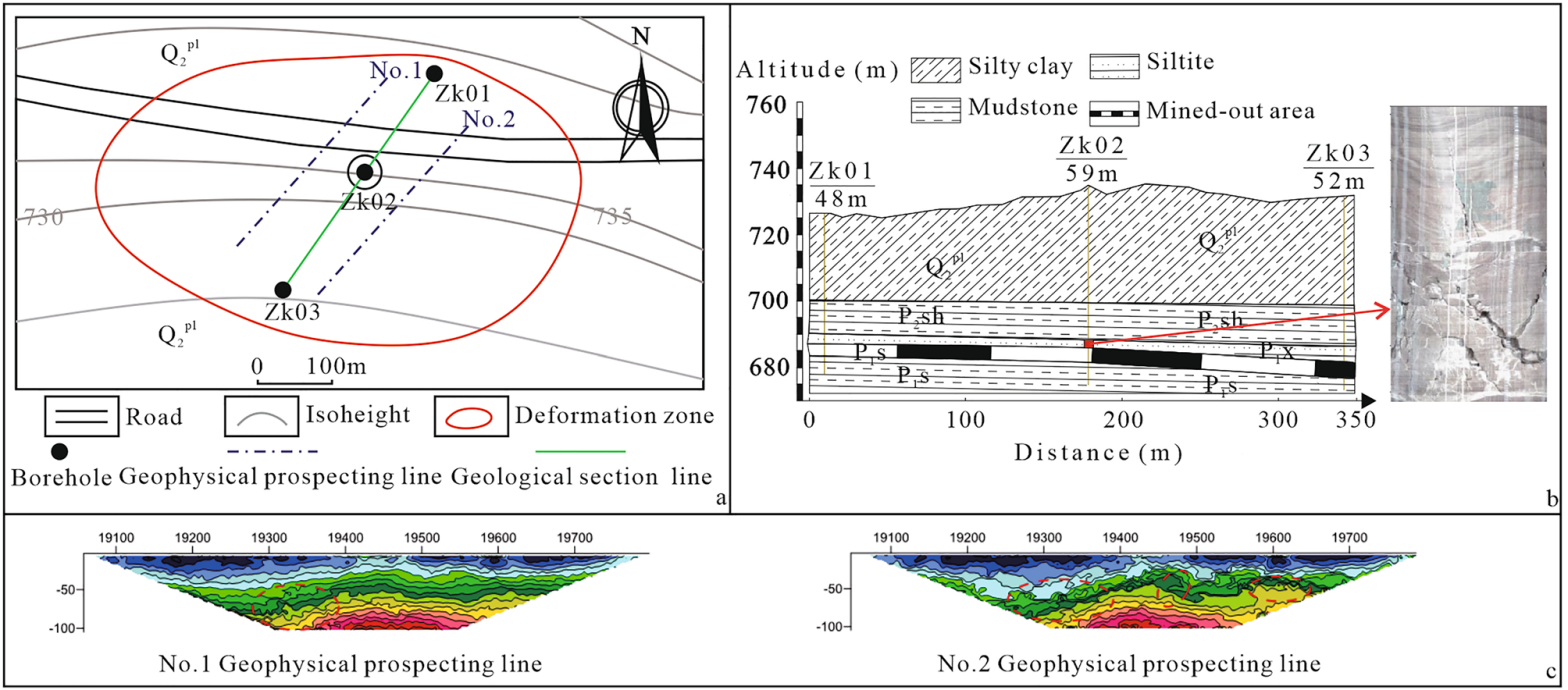
Geophysical exploration and geotechnical exploration results.

The high-density direct current electrical method^33^ was used to survey the deformation area. The geophysical prospecting lines are arranged 60 m on both sides of the maximum deformation point obtained by SBAS-InSAR. A total of 2 measuring lines are laid, 300 m for each measuring line, spacing of measuring points of 5 m, and 120 measuring points (Fig. 3a).

Combined with the SBAS-InSAR results and geophysical prospecting results, three boreholes were arranged in the deformed area. Boreholes No. 1 and No. 3 occur near the edge of the deformation area, and Borehole No. 2 is located at the position of maximum deformation according to the SBAS-InSAR results. The three boreholes are in a straight line. The geological structure of the main deformation area was revealed by drilling, and the integrity of the mined-out area roof was observed by borehole TV technology^34^ (Fig. 3a).

On this basis, we establish a physical model to conduct a physical simulation test^35,36^, and the deformation mechanism of the overlying strata in the mined-out area of the Lanhe Coal Mine is analysed.

## Results analysis

### InSAR

The InSAR monitoring results include interference results and deformation results. After a series of steps, such as image registration, interferogram generation, removal of the flat phase and terrain phase, and smoothing, all interferogram images are obtained. The final satellite line-of-sight deformation result of the surface is obtained after the interpretation of the interference image. According to the geometric relationship of the image, the image is reprojected (Fig. 2a) and converted into the vertical deformation result (Fig. 2b) (Liu et al.^32^).

The temporal deformation results of Points P1 to P8 in the region are extracted to form the temporal deformation diagram of each point (Fig. 2c). Each point has obvious deformation from September 2018 to September 2020. The deformation process and characteristics at each time stage of the region are described as follows. (1) From September 2018 to January 2019, the regional deformation was relatively slow and in the slow deformation stage. (2) From February 2019 to April 2020, large deformation occurred in the region, but the deformation rate was relatively stable, and the regional deformation was in the uniform deformation stage. (3) After May 2020, the overall deformation rate of the region increased significantly, and the regional deformation was in the accelerated deformation stage.

To further analyse the deformation characteristics of the surface deformation area, the profile line is selected in the deformation area (Fig. 2b). The time series cumulative deformation at the end of the three deformation stages was extracted to draw the surface deformation profile of the mined-out area (Fig. 2d), and the deformation area was analysed in the profile. (1) In the slow deformation stage, the surface subsidence occurs uniformly, and the maximum subsidence is 48 mm. (2) In the uniform deformation stage, large surface deformation occurs, and a subsidence basin is formed. The deformation of each point on the section is quite different, and the maximum subsidence is 290 mm. (3) In the accelerated deformation stage, the surface subsidence basin is further developed, and the basin shape is similar to that in the previous stage, with a maximum subsidence of 430.3 mm.

The deformation stage and deformation area obtained by InSAR are basically consistent with the results obtained through field investigations.

### Geophysical prospecting and geotechnical exploration

In the quasisectional view of the apparent resistivity of the detection line of object No. 1 (Fig. 3c), the resistivity curve of the shallow part is relatively gentle, reflecting the relatively uniform and complete structure of the shallow stratum. At a burial depth of approximately 60 m on the left side, the apparent resistivity curve has local distortion deformation, which shows a local high-resistance anomaly. In the quasisectional view of the apparent resistivity of the detection line of object No. 2 (Fig. 3c), the local distortion of the apparent resistivity curve appears at burial depths of approximately 50–60 m, which is manifested as a local high-resistivity anomaly. Combined with the geological data of the survey area, it is speculated that the abnormal area is a high-resistivity abnormal area caused by the mined-out area, and the burial depth is approximately 50–60 m.

**Figure 4:**
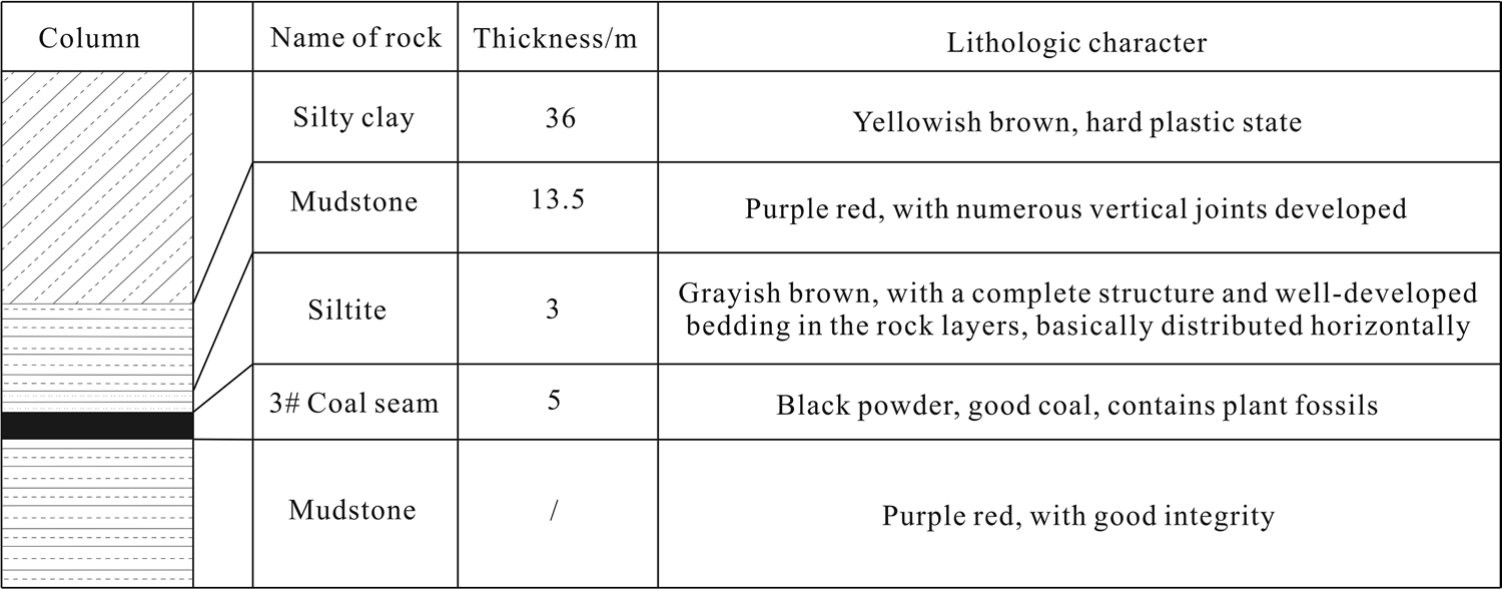
Typical engineering geological histogram of the study area.

The borehole shows that the geological structure of the surface deformation region from top to bottom is as follows. (1) The Middle and Pleistocene Quaternary Lishi Formation silty clay is 25–36 m thick and has a hard plastic state with upper cracks. (2) The purplish red mudstone of the Upper Permian Shiqianfeng Formation is approximately 9.8–13.5 m thick. Affected by early diagenesis and tectonic movements, many vertical joints are developed in the mudstone. (3) The siltite of the Lower Permian Lower Shihezi Formation comprises the roof in the mined-out area with a thickness of approximately 2.8–3.6 m. The siltite structure is complete with high integrity, and joints and fractures are poorly developed. The bedding development in the rock layer is obvious, and the bedding is basically horizontal. (4) The thickness of coal seam #3 of the lower Permian Shanxi Formation is approximately 4.3–5 m. (5) The floor of the mined-out area is mudstone of the lower Permian Shanxi Formation (Figs. 3b, 4).

To ensure the smooth progress of the drilling work, the casing pipe extends to the bottom of the mudstone during the drilling process. When drilling to the mudstone layer, the drilling circulating fluid starts to leak and continues downwards, and the circulation fluid leakage is severe until the mudstone layer is crossed. In addition, leakage is serious in the middle strata of mudstone and at the junction of mudstone and siltstone. This indicates that the mudstone layer has undergone bending deformation, and the rock layer has developed many separation fissures. Large-scale separation fissures are developed between the mudstone and siltstone, or the mudstone fragmentation body is very loose.

In Borehole No. 1, the drill pipe dropped 4.3 m at the bottom of the siltstone layer, which exposed the mined-out area. In Borehole No. 2, the drill pipe dropped 4.7 m at the bottom of the siltstone layer, which exposed the mined-out area. In Borehole No. 3, the drill pipe did not drop, which exposes the coal pillar.

After drilling, the roof integrity and the mined-out area status were observed by Borehole TV. According to the Borehole TV results, the roofs of the mined-out areas of Boreholes No. 1 and No. 3 are complete, and there are cracks and fracture zones in the siltstone of the roof of Borehole No. 2 (Fig. 3b). This does not reveal the shattered zone, indicating that the roof is ruptured, and cracks develop, but no caving occurs.

Based on geophysical prospecting and geotechnical exploration results, the following conclusions can be drawn. (1) There is a large area of shallow room and pillar mined-out areas under the deformation area, and the coal pillar in the mined-out area partially fails. However, the roof integrity is good and does not collapse. (2) Due to the large deflection of the mined-out area roof below the maximum point of surface deformation, which exceeds the ceiling deformation limit, cracks and fracture zones are developed, and the overburden rock of the mined-out area is obviously bent. (3) The deformation of the roof leads to the large deformation of the overlying mudstone layer, which is in a relatively broken state. The deformation of the mudstone layer creates space for the deformation of the unconsolidated formation, resulting in surface subsidence and the development of cracks.

### Physical model experiments

#### Physical model design

The physical model experiment must satisfy the principles of similarity theory, which can ensure that the indoor model can simulate the actual engineering situation more accurately. In this paper, the experiment is conducted under a self-gravity stress field. Therefore, the main similarity constants include the geometric similarity constants *C*_L_ and density similarity constants *C*_*ρ*_.

1 $$ C_{{\text{L}}} = L_{{\text{p}}} /L_{{\text{m}}} $$

2 $$ C_{\rho } = \rho_{{\text{p}}} /\rho_{{\text{m}}} $$

In Eqs. (1) and (2), *L*_p_ is the size of the prototype, *L*_m_ is the size of the model, *ρ*_p_ is the density of the prototype, and *ρ*_m_ is the density of the model.

**Figure 5:**
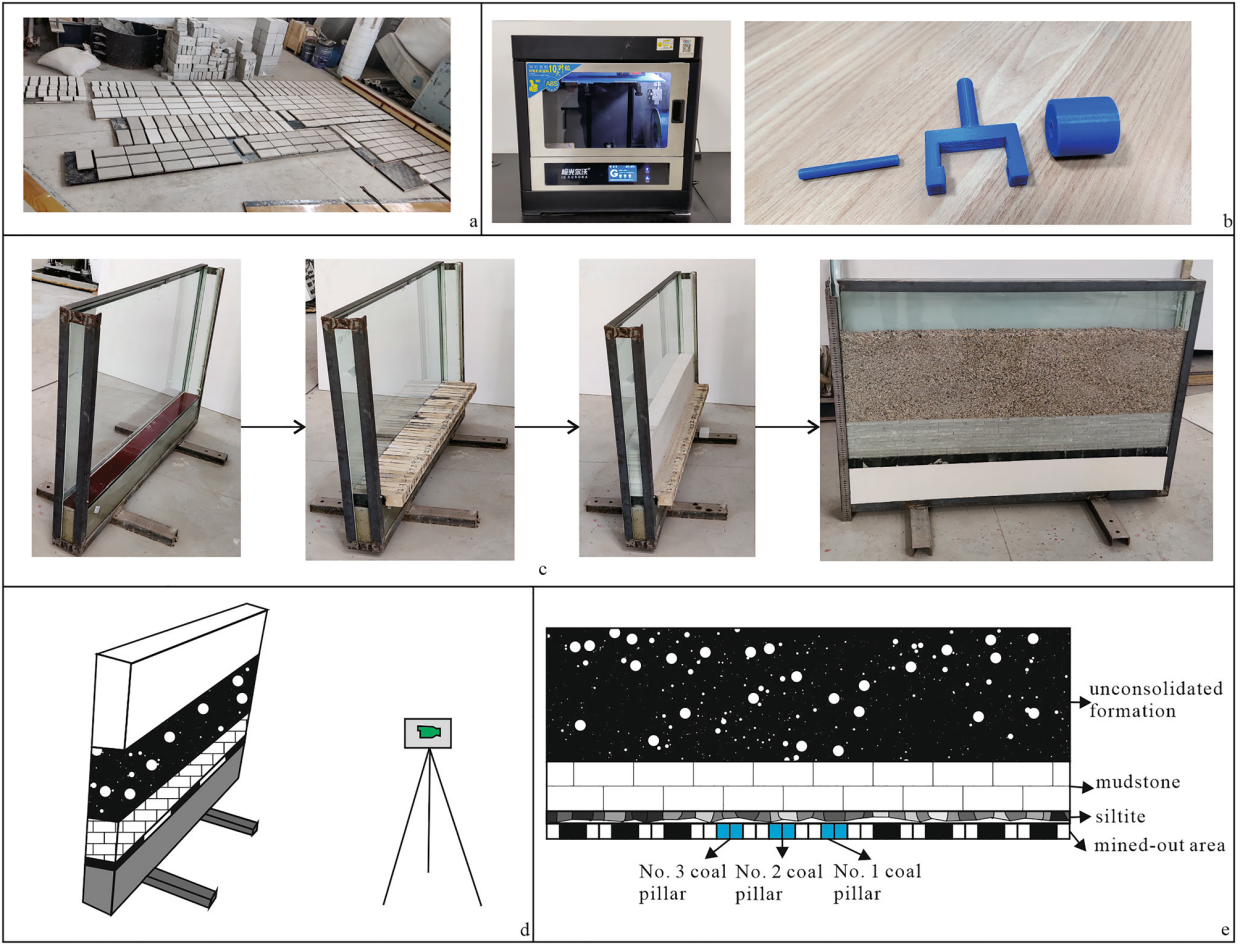
Physical model test design.

In this physical model test, the geometric similarity constant is 100, and the density similarity constant is 1.25. Since the deformation characteristics of the overlying strata in the case of small deformation of the roof before fracture and collapse are the only focus of this study, to avoid the interference caused by complex simulation materials of the overlying strata, the rock strata are simulated with prefabricated panels of similar materials, and the silty clay is simulated with gravel, which is easy to monitor by particle image velocimetry technology. To prevent gravel sliding along the gap between the prefabricated plate and the model box from affecting the test, the gravel particle size of the simulated unconsolidated formation was selected as 5–10 mm (Fig. 5c). The coal seam is simulated by 40 blocks of 3.5 cm width and 5 cm height. The length of the blocks is 20 cm because it is easy to extract to simulate coal seam mining. In addition, to highlight the appearance of the coal seam, black paint was applied to the end face and side parts of the wood block (Fig. 5c).

Rock layer model materials usually comprise different materials mixed in a certain ratio. The main mineral component of river sand is SiO_2_, which exhibits characteristics such as high hardness, high wear resistance, and stable chemical properties. It imposes a certain regulatory effect on the strength and elastic modulus of similar materials and can overcome the shortcomings of high impurity content, complex grading, and poor cementitious bonding among ordinary sand particles. It can be used as an aggregate for similar materials. Barite powder exhibits a high bulk density, low strength and elastic modulus, and stable performance at high temperatures, rendering it an ideal material for adjusting the mechanical properties of similar models. After hardening, gypsum exhibits significant brittleness, low apparent density, and low strength, so it is a suitable bonding agent for similar materials. Therefore, the siltstone and mudstone in this physical simulation experiment were simulated using a mixture of gypsum, barite powder, river sand, and water. To meet the experimental requirements of the mixing ratio and rock layer scheme, a series of indoor experiments were conducted, and the mixing ratio and model parameters were accurately selected and calculated. The specific mixing ratio is provided in Table 1.

**Table 1:** Rock simulation material ratio.

Rock types	Water	Barite powder	River sand	Gesso
Mudstone	1	1	1	1
Siltite	0.8	1	1	1.2

To ensure the effect of the physical simulation test and facilitate the development of the model test, blocks with a length of 200 cm, a width of 10 cm and a height of 1 cm were made, and the blocks were layered and miscoded to simulate the mudstone layer^37^. Blocks with a length of 140 cm, a width of 10 cm and a height of 1 cm were made, and the block was layered to simulate the siltstone layer (Fig. 5a,c).

The dimensions of the physical model are 140 cm × 10 cm × 100 cm (length × width × height), which correspond to an actual engineering scope of 140 m × 10 m × 100 m. According to the actual situation of the strata in the study area, the thicknesses of the coal seam, siltstone layer, mudstone layer and clay layer in the model are set to 5 cm, 3 cm, 13.5 cm and 36 mm, respectively (Fig. 5c,e).

Before the physical model test, a 3D printer was used to produce a small roller (Fig. 5b). The small roller was coated with a convex granular rubber film, and black spots were uniformly painted on the side of the plate after dipping the oil to facilitate particle image recognition and monitor the deformation of rock layers. In the model experiment, a camera was used to obtain real-time photos of the front of the physical model (Fig. 5d). After the experiment, PIV software was used to postprocess the images and calculate the displacement changes of the same particle at different stages to obtain information on the displacement field changes in the loose layer and overlying rock. The spatial resolution of the monitoring measurements is 0.02 mm.

**Figure 6:**
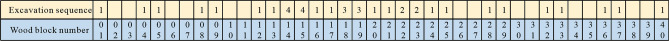
Mining scheme.

According to the characteristics of the room and pillar mined-out area in the study area, the mining scheme of this physical model experiment is to excavate a 7 cm coal seam and set a 7 cm coal pillar. The mining is conducted until the mining rate is 50%, and then wood blocks are extracted successively to simulate the instability of the coal pillar (Fig. 5e). The specific excavation plan is summarized in Table 2 and Fig. 6.

**Table 2:** Excavation sequence and simulation conditions.

Excavation sequence	Simulation conditions
1	Recovery rate 50%
2	#1 coal pillar instability
3	#2 coal pillar instability
4	#3 coal pillar instability

#### Experimental result analysis

**Figure 7:**
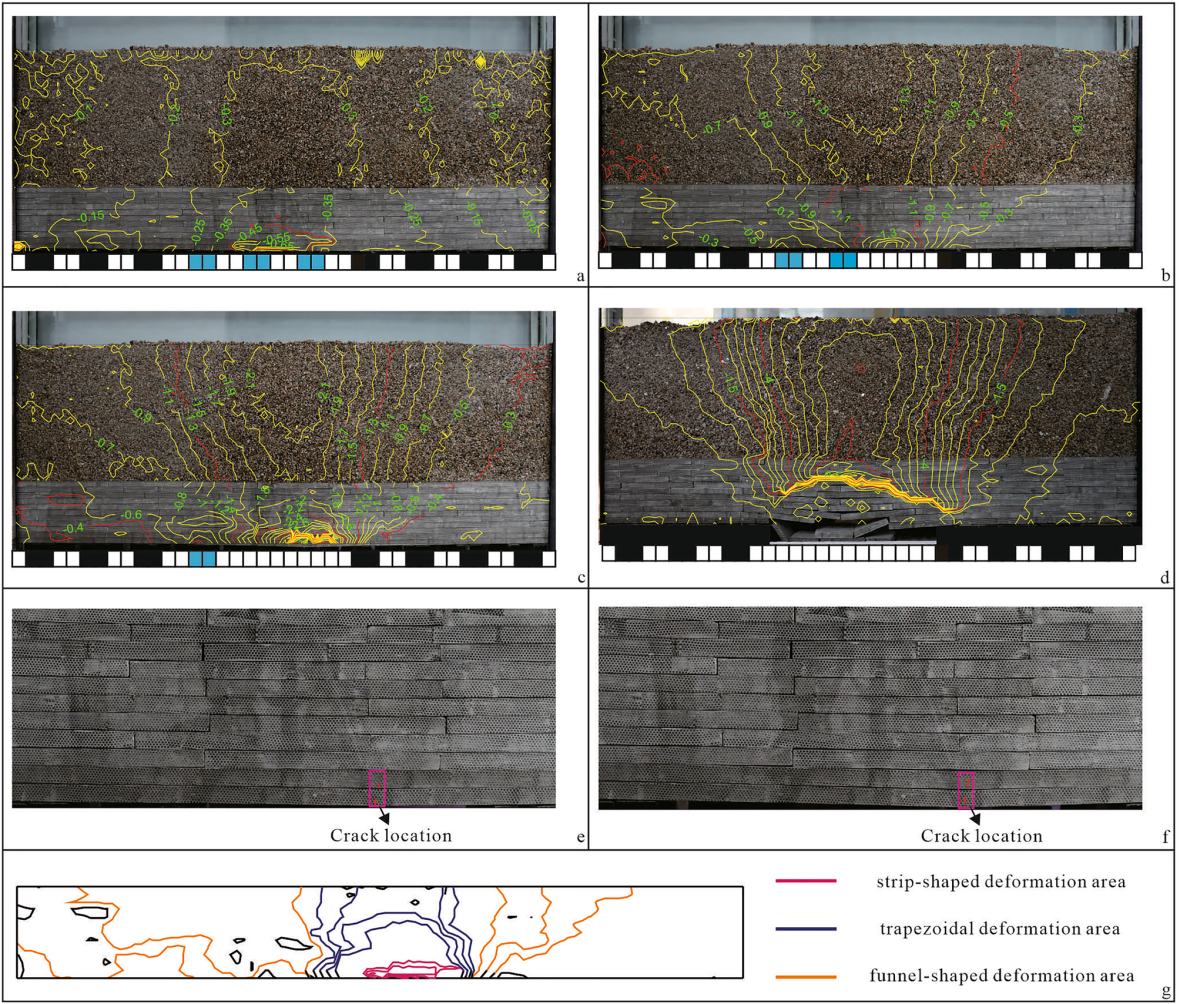
Model test results.

At the end of room and pillar mining, the roof has undergone bending deformation. The maximum deformation location is the roof of the central mined-out area of the model. The maximum subsidence of the roof is 0.65 mm (Fig. 7a). The deformation of the unconsolidated formation is trapezoidal. The deformation develops to the surface, but the magnitude is small.

The unloading effect of coal seam mining causes the deformation of overlying rock and the overlying unconsolidated formation, but the overlying rock is in a stable state and does not collapse. Thus, it can play a better supporting role for the overlying unconsolidated formation. The moving deformation of the overburden creates space for the deformation of the unconsolidated formation (Fig. 7a).

After the instability of the No. 1 coal pillar, the roof deformation increases. The maximum deformation position is the roof above the No. 1 coal pillar. The maximum subsidence reaches 1.5 mm. Cracks develop in the roof, and the overburden begins to produce separation cracks (Fig. 7b,e), with a maximum deformation of 1.3 mm at the surface. After the instability of the No. 2 coal pillar, separation cracks continue to develop. The roof deformation above the No. 1 coal pillar continues to increase. The cracks extend upwards, but they do not penetrate the rock layer (Fig. 7c,f), resulting in a maximum surface deformation of 2.1 mm. After the instability of the No. 3 coal pillar, the roof of the mined-out area is broken and collapsed, and the crack between the layers expands (Fig. 7d), with the maximum surface deformation reaching approximately 7 mm. After the occurrence of coal pillar instability, the surface deformation before and after roof failure is of the same magnitude.

After the instability of the No. 2 coal pillar occurs, the state of the roof of the physical model mined-out area is consistent with the actual situation in the field. At this time, the mined-out area roof is in the limit equilibrium state. The continued instability of the coal pillar will lead to roof failure and collapse. At the same time, the deformation of the unconsolidated formation shows a funnel shape, and the maximum subsidence in the centre of the surface reaches 2.1 mm. This indicates that under the geological structure conditions of the study area, the small deformation of the roof before fracture and collapse caused by coal pillar instability has a great influence on the deformation of the overlying strata in the mined-out area, which can lead to a great deformation of the surface and a great influence on the surface production activities.

In the following, we summarize the deformation characteristics of the overlying strata in room and pillar mined-out areas.

1. At the different deformation stages of the mined-out area, the overlying rock deformation can be divided into three deformation areas: strip-shaped deformation area, trapezoidal deformation area and inverted funnel-shaped deformation area. Among them, the strip-shaped deformation area develops at the maximum deflection of the roof, and the deformation is the most notable. Thus, the size and deformation magnitude of the overburden rock in other deformation areas are controlled. Due to the thin overburden, the overburden deformation near the deformation centre is trapezoidal, while the overburden deformation far away from the deformation centre exhibits an inverted funnel shape. After the completion of room and pillar mining, strip-shaped and trapezoidal deformation areas are mainly developed in the overlying rock. With the occurrence of coal pillar instability, trapezoidal and inverted funnel-shaped deformation areas are largely developed in the overlying rock (Fig. 7g).
2. The deformation characteristics of unconsolidated formations develop from a trapezoidal shape after room and pillar mining to a funnel shape after coal pillar instability occurs. With the increase in the unstable coal pillar, the deformation of the unconsolidated formation increases, and the funnel-shaped deformation area gradually expands from the centre to both ends. The maximum deformation point of the surface is located in the centre of the funnel, which is consistent with the vertical direction of the rock crack development.

Based on comprehensive analysis of the physical simulation results and InSAR monitoring results, it could be concluded that after the completion of room and pillar mining, roof deformation caused minor surface deformation, while long-term coal pillar instability did not occur, and the surface remained stable from September 2018 to January 2019. From February 2019 to April 2020, the coal pillar in the mined-out area experienced local instability, and the roof experienced significant deformation, which was transmitted to the surface. After May 2020, the number of unstable underground coal pillars continued to increase, and increasing surface deformation was developed. At this time, the roof occurred in a critical state before failure.

## Discussion

### Analysis of the failure mechanism of the overlying rock above the room and pillar mined-out area

**Figure 8:**
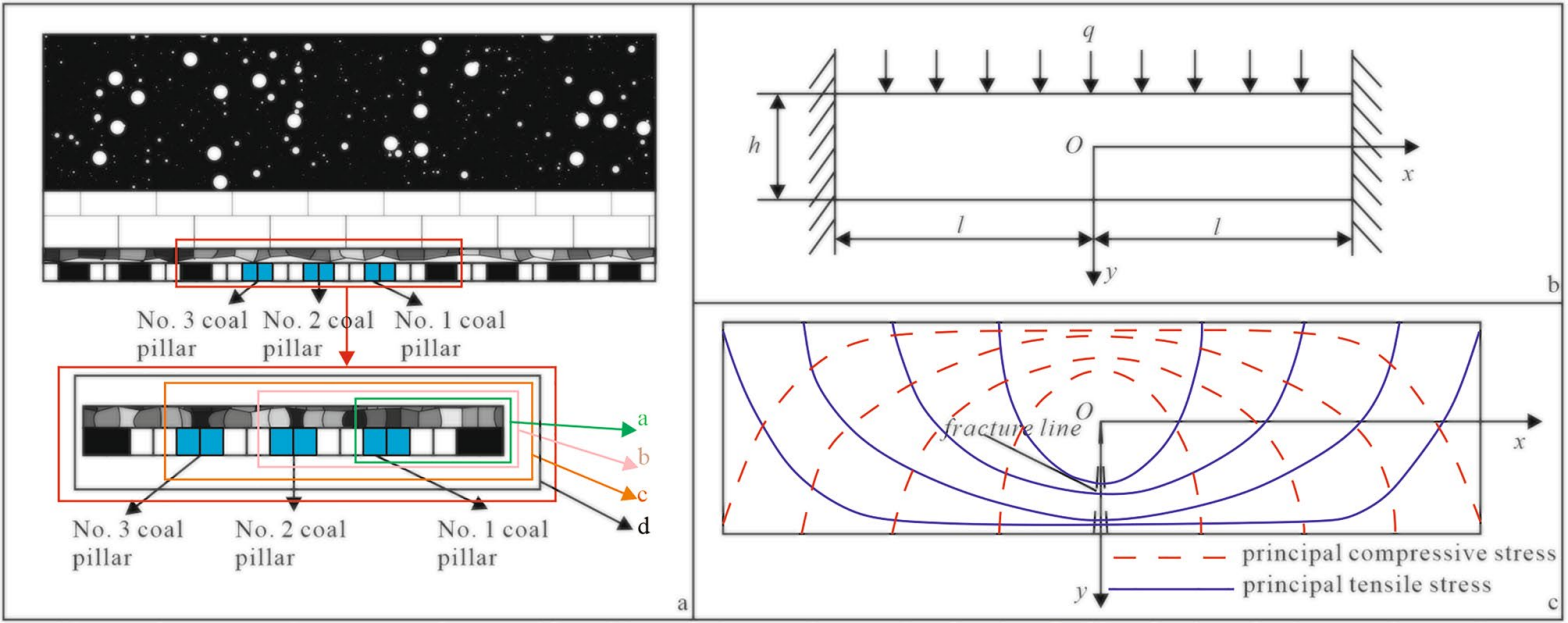
Mechanical model of the roof and stress trace distribution^13^.

After room and pillar coal mining, the coal pillar roof system is shown in Fig. 8a(a), which can be regarded as a fixed beam model under a uniformly distributed load (Fig. 8b). The expression of the stress component at any point in the fixed supported beam is shown in Eq. (3)^13^.

3 $$ \begin{gathered} \sigma_{x} = \frac{6qy}{{h^{3} }}(l^{2} - x^{2} ) + \frac{qy}{h}\left( {\frac{{4y^{2} }}{{h^{2} }} - \frac{3}{5}} \right) \hfill \\ \sigma_{y} = - \frac{q}{2}\left( {1 + \frac{y}{h}} \right)\left( {1 - \frac{2y}{h}} \right)^{2} \hfill \\ \tau_{xy} = - \frac{6qx}{{h^{3} }}\left( {\frac{{h^{2} }}{4} - y^{2} } \right) \hfill \\ \end{gathered} $$

where the load borne by each rock layer in bedrock can be calculated by Eq. (4)^13^.

4 $$ (q_{n} )_{1} = \frac{{E_{1} h_{1}^{3} (\gamma_{1} h_{1} + \gamma_{2} h_{2} + \cdots + \gamma_{n} h_{n} )}}{{E_{1} h_{1}^{3} + E_{2} h_{2}^{3} + \cdots + E_{n} h_{n}^{3} }} $$

In Eq. (4), *E*_n_ is the elastic modulus of the n-th layer above the rock layer in the bedrock, MPa; *h*_n_ is the thickness of the n-th layer above the rock layer in the bedrock, m; and *γ*_n_ is the bulk density of the n-th layer above the rock layer in the bedrock, kN/m^3^.

Because the roof strata are compressive but not tensile, the roof beam will undergo tensile failure along the direction perpendicular to the main tensile stress. According to the relationship between the principal stress and stress components, the expressions of the principal tensile stress *σ*_1_ and principal compressive stress *σ*_3_ at any point on a beam can be obtained^13^.

5 $$ \begin{gathered} \sigma_{1} = \frac{{\sigma_{x} + \sigma_{y} }}{2} + \sqrt {\left( {\frac{{\sigma_{x} - \sigma_{y} }}{2}} \right)^{2} + \tau_{xy}^{2} } \hfill \\ \sigma_{3} = \frac{{\sigma_{x} + \sigma_{y} }}{2} - \sqrt {\left( {\frac{{\sigma_{x} - \sigma_{y} }}{2}} \right)^{2} + \tau_{xy}^{2} } \hfill \\ \end{gathered} $$

In Eq. (5), *σ*_1_ is the principal tensile stress. *σ*_3_ is the principal compressive stress.

Combined with Eqs. (3) and (5), the distribution law of principal tensile stress *σ*_1_ and principal compressive stress *σ*_3_ in the fixed rock beam when the roof is fractured for the first time can be obtained (Fig. 8c). For a fixed beam under uniform load, the principal tensile stress *σ*_1_ and principal compressive stress *σ*_3_ are symmetrically distributed in the neutral axis. The principal stress traces near the upper and lower edges of the middle of the beam are approximately horizontal. The principal tensile stress *σ*_1_ and principal compressive stress *σ*_3_ at any point are perpendicular to each other.

According to the theory of the maximum tensile stress, the main factor for brittle materials that fracture is the maximum principal tensile stress. The roof beam will have tensile failure along the direction perpendicular to the principal tensile stress. According to the distribution law of principal tensile stress, the fracture trajectory of the rock beam can be predicted (Fig. 8c). After the instability of the No. 1 coal pillar (Fig. 8a(b)), vertical cracks are generated in the tensile stress zone across the middle and lower surfaces and extend vertically upwards. After the instability of the No. 2 coal pillar (Fig. 8a(c)), the mid-span position changes. However, at this time, the rock beam is damaged along the weak surface of cracks. The original vertical cracks continue to expand, and fracture almost begins to occur. The roof is in the limit equilibrium state. After the instability of the No. 3 coal pillar (Fig. 8a(d)), the limit equilibrium state of the roof was broken, and rock beam shear failure occurred along the weak surface of the fracture.

Compared to those of the bedrock, the tensile, shear and bending abilities of the unconsolidated formation are very low. After the completion of the room and pillar mining, the bedrock deformation extends to the interface, and the lower loose layer undergoes sliding failure at the edge of the mined-out area, leading to tensile failure of the upper near-surface loose layer and slight surface subsidence. Under the condition of coal pillar instability, sliding failure and tensile failure gradually develop and accumulate. The shear stress imposed on the middle loose layer gradually reaches its limit, and shear failure occurs. When the roof deformation occurs in the ultimate equilibrium state, the maximum surface settlement deformation reaches 2.1 mm. At this point, the loose layer gradually undergoes slip tension shear failure at a certain angle with the horizontal plane, forming a funnel-shaped failure mode.

### Analysis of the linkage failure process of the loose layer and overburden rock

Based on the physical simulation experimental results, in this article, we can describe the evolution process of loose layer overlying rock failure under deformation and instability conditions in shallow and gently inclined mined-out areas as follows: ① before coal seam mining, all static loads generated by the self-weight of the loose layer act on the bedrock and occur in a static equilibrium state. ② After the completion of room and pillar mining, the original static balance is disrupted, and stress concentration occurs in the roof at the middle of the mined-out area, causing deformation of the overlying rock in the mined-out area and synchronous and coordinated settlement of the overlying loose layer. The deformation of the loose layer develops into a trapezoidal shape. ③ Under the condition of coal pillar instability, the deformation of the loose layer transitions from a trapezoidal shape to a funnel shape. Due to the nature of the loose layer, it cannot provide a stable bearing structure, and the various media in the transport area still act on the bedrock in the form of loads, further exacerbating the development of overlying rock deformation in the mined-out area. ④ The intensified deformation of the overlying rock, in turn, leads to further settlement and migration of the loose layer, thereby expanding the funnel-shaped damage area and inducing a new equilibrium state. ⑤ The coal pillar continues to lose stability, and cracks develop in the roof, which eventually reaches the ultimate equilibrium state.

In summary, it can be observed that the static load caused by the loose layer is the fundamental driving force of the deformation of the overlying rock, and the mechanical characteristics of the loose layer and overlying rock, as well as the joint evolution of these two media, are the fundamental reasons for the unique deformation mechanism.

The maximum deformation point at the surface in the room and pillar mined-out area after coal pillar instability corresponds to the position of the initial unstable coal pillar and the crack in the mined-out area roof along the vertical direction. We can approximately determine the position of the initial unstable coal pillar in the mined-out area through the maximum deformation point at the surface and conduct refined surveys near this position using drilling, borehole geophysical exploration, and other methods to determine the position of the unstable coal pillar. Through grouting, separation cracks in the overlying rock and the mined-out area can be filled, thereby controlling the stability of the mined-out area and effectively preventing further development of deformation of the overlying strata above the cavity in the mined-out area.

## Conclusions

In this paper, a case of overlying strata deformation in a mined-out area caused by coal pillar instability in Jincheng, Shanxi Province, is studied by using various methods. The deformation development mechanism of the roof, overburden, unconsolidated formation and surface in the mined-out area is analysed, and the following conclusions can be obtained:

1. The surface deformation caused by coal pillar instability in the room and pillar mined-out area exhibits the slow deformation stage, uniform deformation stage and accelerated deformation stage. The maximum deformation point at the surface corresponds to the position of the initial coal pillar instability and the crack in the mined-out area roof along the vertical direction.
2. After the completion of room and pillar mining, strip-shaped and trapezoidal deformation areas are mainly developed in the overlying rock. With the occurrence of the coal pillar instability, trapezoidal and inverted funnel-shaped deformation areas are primarily developed in the overlying rock. The deformation characteristics of unconsolidated formations transition from trapezoidal deformation after room and pillar mining to funnel-shaped deformation after the occurrence of coal pillar instability. Moreover, the maximum surface deformation point is located at the centre of the funnel.
3. The mined-out area roof can be regarded as a fixed beam model under a uniform load before fracturing. Under the condition of coal pillar instability, vertical cracks can occur in the tensile stress zone of the middle and lower surfaces of the rock beam span and expand vertically upwards. Continuous coal pillar instability can lead to the failure of the rock beam along the weak surface of the original crack and finally result in tensile fractures of the rock beam. When the bedrock deformation extends to the interface, the unconsolidated formation sequentially experiences slippage, tension fracture and shear failure and finally forms the funnel-shaped failure mode.
4. In this article, we explore mined-out area treatment methods based on the deformation characteristics of the overlying strata in the room and pillar mined-out area under the condition of coal pillar instability. However, this article performs two-dimensional physical simulation experiments, which exhibit certain limitations. Three-dimensional model experiments can be used to accurately reflect the surface deformation morphology in mined-out areas and constitute the development trend of research on the deformation mechanism of overlying rock in mined-out areas.

## Data Availability

Thank you very much for the attention to our research. The datasets generated and analysed during the current study are not publicly available due to the company’s confidentiality requirements for the raw data but are available from the corresponding author on reasonable request.

## References

[1] Wang X, Yang T, Guan K, Liu X, Zhao Y (2022). Stability evaluation of multi-pillar and roof system based on instability theory. Rock Mech. Rock Eng..

[2] Luo R, Li G, Chen L, Yang Q, Zang C, Cao W (2020). Ground subsidence induced by pillar deterioration in abandoned mine districts. J. Cent. South Univ..

[3] Henry SH, Robert JW, Don JG (1989). Induced seismicity in mines in Canada: An overview. PAGEOPH.

[4] Roger JM, Chugh YP, Roscetti T (1986). Subsidence prediction in shallow room and pillar mines. Int. J. Min. Geol. Eng..

[5] Zhu W, Xu J, Chen L, Li Z, Liu W (2019). Mechanism of disaster induced by dynamic instability of coal pillar group in room-and-pillar mining of shallow and close coal seams. J. China Coal Soc..

[6] Shen, B., Alehossein, H., Poulsen, B. A.. Collingwood park mine remediation-subsidence control using fly ash backfilling. *CSIRO Earth Science and Resource Engineering Report EP105068*. (2010).

[7] Shen B, Poulsen BA, Luo X (2017). Remediation and monitoring of abandoned mines. Int. J. Min. Sci. Technol..

[8] Ju J, Xu J (2013). Structural characteristics of key strata and strata behaviour of a fully mechanized longwall face with 7.0m height chocks. Int. J. Rock Mech. Min. Sci..

[9] Xu J, Qian M, Zhu W (2005). Study on influences of primary key stratum on surface dynamic subsidence. Chin. J. Rock Mech. Eng..

[10] Zhu W, Xu J, Shi X, Wang X, Liu W (2009). Research on influence of overburden primary key stratum movement on surface subsidence with in-situ drilling test. Chin. J. Rock Mech. Eng..

[11] Wang B, Xu J, Xuan D (2018). Time function model of dynamic surface subsidence assessment of grout-injected overburden of a coal mine. Int. J. Rock Mech. Min. Sci..

[12] Wang F, Xu J, Xie J (2019). Effect of arch structure in unconsolidated layers on fracture and failure of overlying strata. Int. J. Rock Mech. Min. Sci..

[13] Zuo J, Sun Y, Qian M (2017). Movement mechanism and analogous hyperbola model of overlying strata with thick alluvium. J. China Coal Soc..

[14] Sun Y, Zuo J, Karakus M, Wen J (2019). Investigation of movement and damage of integral overburden during shallow coal seam mining. Int. J. Rock Mech. Min. Sci..

[15] Sun Y, Zuo J, Karakus M, Wen J (2020). A novel method for predicting movement and damage of overburden caused by shallow coal mining. Rock Mech. Rock Eng..

[16] Bell FG, de Bruyn IA (1999). Subsidence problems due to abandoned pillar workings in coal seams. Bull. Eng. Geol. Env..

[17] Fakhimi A, Hosseini O, Theodore R (2016). Physical and numerical study of strain burst of mine pillars. Comput. Geotech..

[18] Kelly M, Luo X, Craig S (2022). Integrating tools for longwall geomechanics assessment. Int. J. Rock Mech. Min. Sci..

[19] Unlu T, Akcin H, Yilmaz O (2013). An integrated approach for the prediction of subsidence for coal mining basins. Eng. Geol..

[20] Salmi EF, Nazem M, Karakus M (2017). The effect of rock mass gradual deterioration on the mechanism of post-mining subsidence over shallow abandoned coal mines. Int. J. Rock Mech. Min. Sci..

[21] Ghabraie B, Ren G, Barbato J (2017). A predictive methodology for multi-seam mining induced subsidence. Int. J. Rock Mech. Min. Sci..

[22] Qin Y, Xu N, Zhang Z (2016). Failure process of rock strata due to multi-seam coal mining: insights from physical modelling. Rock Mech. Rock Eng..

[23] Zhang Z, Deng M, Wang X (2020). Field and numerical investigations on the lower coal seam entry failure analysis under the remnant pillar. Eng. Fail. Anal..

[24] Zhou Y, Feng S, Li J (2020). Study on the failure mechanism of rock mass around a mined-out area above a highway tunnel-similarity model test and numerical analysis. Tunn. Undergr. Space Technol..

[25] Xu H, Lai X, Shan P (2023). Energy dissimilation characteristics and shock mechanism of coal-rock mass induced in steeply-inclined mining: comparison based on physical simulation and numerical calculation. Acta Geotech..

[26] Li X, Li X, Wang Y, Peng W, Fan X, Cao Z, Liu R (2023). The seepage evolution mechanism of variable mass of broken rock in karst collapse column under the influence of mining stress. Geofluids.

[27] Zhang P, Sun C, Fan X, Li S, Wang L, Cao Z (2023). Temporal and spatial evolution mechanisms of the water-conducting fractured zone of overlying strata in the Kongzhuang coal mine. Geofluids.

[28] Wang L, Xue Y, Cao Z, Wu X, Dang F, Liu R (2023). Mechanical properties of high-temperature granite under liquid nitrogen cooling. Geofluids.

[29] Yang Y, Li Q, Liu S, Zhang K, Zhao Y, Zhang G, Zhang W, Liu G (2023). Evolution mechanism of thickly sticky loose layered arch structure and prediction method of initial ground fissure caused by underground coal mining. Geofluids.

[30] Yang Y, Li Y, Wang L, Wu Y (2023). On strata damage and stress disturbance induced by coal mining based on physical similarity simulation experiments. Sci. Rep-UK..

[31] Cao Z, Du F, Xu P, Lin H, Xue Y, Zhou Y (2017). Control mechanism of surface subsidence and overburden movement in backfilling mining based on laminated plate theory. Cmc-Comput. Mater. Contin..

[32] Liu Z, Mei G, Sun Y, Xu N (2021). Investigating mining-induced surface subsidence and potential damages based on SBAS-InSAR monitoring and GIS techniques: a case study. Environ. Earth Sci..

[33] Wu G, Yang G, Tan H (2016). Mapping coalmine goaf using transient electromagnetic method and high density resistivity method in Ordos City, China. Geod. Geodyn..

[34] Xu N, Dai F, Li B, Zhu Y, Zhao T, Yang D (2017). Comprehensive evaluation of excavation-damaged zones in the deep underground caverns of the Houziyan hydropower station, Southwest China. Bull. Eng. Geol. Environ..

[35] Kong D, Li Q, Wu G, Song G (2021). Characteristics and control technology of face-end roof leaks subjected to repeated mining in close-distance coal seams. Bull. Eng. Geol. Environ..

[36] Qin Y, Xu N, Zhang Z, Zhang B (2021). Failure process of rock strata due to multi-seam coal mining: insights from physical modelling. Rock Mech. Rock Eng..

[37] Mandl G (2005). Rock Joints-the Mechanical Genesis.

